# 

**Published:** 1973-04

**Authors:** 


Bristol Medico-Chirurgical Journal

(Incorporating the Medical Journal of the South-West)

Established 1883

Vol. 88 (ii) APRIL 1973 No. 326

### BRISTOL MEDICO-CHIRURGICAL JOURNAL

Editor: N. J. Brown, M.B., F.R.C.P., F.R.C.Path.

Published Quarterly

Annual Subscription £2 Post Free

### BRISTOL MEDICO-CHIRURGICAL SOCIETY

**President 1972-73: Dr. James Macrae**

**President-elect: Dr. R. F. Barbour**

**Honorary Secretary: Dr. I. S. Bailey, 7 Percival Road, Bristol 8.**

**Honorary Treasurer: Dr. Frank Ross, Bristol Royal Infirmary, Bristol 2.**

The Society was founded in 1874. In 1883 publication of the Journal began and has continued quarterly ever since then. During the years 1949 to 1962 it appeared under the title of "The Medical Journal of the South West".

### THE BRISTOL MEDICO-CHIRURGICAL JOURNAL

**Editorial Committee**

| | |
|---|---|
| Editor | Dr. N. J. Brown, Southmead Hospital, Bristol |
| Assistant Editor | Dr. D. S. Reeves. |
| Members | Dr. Rhys Davies.<br>Dr. M. B. Lennard.<br>Professor R. C. Wofinden.<br>Dr. D. W. Wright.<br>The Hon. Treasurer and Hon. Secretary of the Society. |
| Business Manager | Dr. P. J. F. Baskett, Frenchay Hospital, Bristol. |
| Secretary | Mr. B. P. Jones, The Library, Medical School, University Walk, Bristol BS8 1TD. |

### NOTICE TO CONTRIBUTORS

The Journal is especially concerned to provide a place for the recording of medical thought, interests, and practice in and around Bristol. It therefore welcomes articles that, as well as being of immediate interest to members, will document the local and contemporary medical scene for our successors.

Original articles are invited provided that they have not been published elsewhere. References in the text should be by author's name, with year of publication in parenthesis; they should be listed at the end in alphabetical order, the name of each journal or book being given in full—not abbreviated—and the title of the article added. Illustrations should be supplied ready for reproduction. Line drawing should be in Indian ink on firm white paper or board. The author will be informed if it is found necessary to ask him to pay for the making of blocks.

The following contributions will be especially welcome; notes of forthcoming events, meetings held, degrees conferred, staff appointments, honours and public appointments and other local news.

### The Medical Library

In 1890 the Bristol Medico-Chirurgical Society founded a medical library which in 1892 was combined with that of the Bristol Medical School. This became the University of Bristol Medical Library in 1925 and an agreement in that year confirmed the privilege of Members of the Society to use the University Medical Library.

### A Selection of Recent Additions to the Medical Library

ADAM, G. ed.: Biology of memory. (Akad. Kiadó)

ALPERS, B. J. & MANCALL, E. L.: Clinical neurology. Ed. 6. (Blackwell)

ALTMAN, P. L. & DITTMER, D. S. eds.: Respiration and circulation (FASEB biological handbook). (Federation of American Societies for Exper, Biol.)

AMOORE, J. E.: Molecular basis of odor. (Thomas)

ANDREW, W.: Anatomy of aging in man and animals. (Heinemann)

ANTONINI, E. & BRUNORI, M.: Hemoglobin and myoglobin in their reactions with ligands. (North-Holland)

BALLANTYNE, A. J. & MICHAELSON, I. C.: Textbook of the fundus of the eye. Ed. 2. (Livingstone)

BARR, W.: Clinical gynaecology. (Churchill Livingstone)

BECK. J. W. & BARRETT-CONNOR, E.: Medical parasitology. (Mosby/Kimpton)

BEERS, R. F. & BRAUN, W. eds.: Biological effects of polynucleotides. (Springer)

BELLANTI, J. A.: Immunology. (Saunders)

BERTELLI, A. & BACK, N. eds.: Shock: biochemical, pharmacological and clinical aspects. (Plenum)

BILLINGHAM, R. & SILVERS, W.: The immunobiology of transplantation. (Prentice-Hall)

BLACK, J. A.: Neonatal emergencies—and other problems. (Butterworth)

BOLEY, S. J. et al.: Vascular disorders of the intestine. (Butterworth)

BOWERS, J. Z. & ROSENHEIM, M. L. eds.: Migration of medical manpower. (Macy Foundation). Presented by Professor D. Russell Davis.

BRIMBLECOMBE, R. W. & PINDER, R. M.: Tremors and tremorogenic agents. (Scientechnica). Presented by John Wright & Sons Ltd.

BRUINING, H. A.: Surgical treatment of hyperparathyroidism, with an analysis of 267 cases. (Royal Van Gorcum)

BRUNSON, J. G. & GALL, E. A. eds.: Concepts of disease: a textbook of human pathology. (Macmillan)

BURDETTE, W. J. ed.: Carcinoma of the colon and antecedent epithelium. (Thomas)

BURLAND, W. L. & LAURANCE, B. M. eds.: The therapeutic choice in paediatrics. (Churchill Livingstone)

BUVET, R. & PONNAMPERUMA, C. eds.: Chemical evolution and the origin of life. (North-Holland)

CAMPS, F. E. & CARPENTER, R. G.: Sudden and unexpected deaths in infancy (cot deaths) (Wright). Presented by John Wright & Sons Ltd.

CHRISTENSEN, H. H.: Neutrality control in the living organism. (Saunders)

KERKUT, G. A. & YORK, B.: The electrogenic sodium pump. (Scientechnica)

KNIGGE, K. M. et al. eds.: Brain-endocrine interaction. Median eminence: structure and function. (Karger)

KREEL, L.: Outline of radiology. (Heinemann)

KUBITSCHEK. H. E.: Introduction to research with continuous cultures. (Prentice-Hall)

LARON, Z.: Habilitation and rehabilitation of juvenile diabetics. (Kroese)

LAVIA, M. F. & HILL, R. B. eds.: Principles of pathobiology. (Oxford Univ. Press)

LENGEROVA, A. & VOJTISKOVA, M.: Immunogenetics of the H-2 system. (Karger)

LINDAHL-KIESSLING, K. et al. eds.: Morphological and functional aspects of immunity. (Plenum Press)

LOVELL, R. R. H. & DOYLE, A. E.: An introduction to clinical medicine. (Melbourne Univ. Press)

LOWE, R. D. & ROBINSON, B. F.: A physiological approach to clinical methods. (Churchill Livingstone)

LUFT, R. & RANDLE, P. J. eds.: On the pathogenesis of diabetes mellitus. Casa Ed. 'Il Ponte'

McLACHLAN, G. ed.: Patient, doctor, society: a symposium of introspections. (Oxford Univ. Press for Nuffield Prov. Hosp. Trust)

MACLEAN, U.: Social and community medicine for students. (Heinemann)

MALMFORS, T. & THOENEN, H. eds.: 6-Hydroxydopamine and catecholamine neurons. (North-Holland)

MEIJNE, N. G.: Hyperbaric oxygen and its clinical value. (Thomas)

MEREDITH, W. J. & MASSEY, J. B.: Fundamental physics of radiology. Ed 2 (Wright). Presented by John Wright & Sons Ltd.

METCALF, D. & MOORE, M. A. S.: Haemopoietic cells. (North-Holland)

MITCHISON, J. M.: The biology of the cell cycle. (Cambridge Univ. Press)

MONTAGNA, W. & BILLINGHAM, R. E.: Immunobiology and the skin. (Appleton-Century-Crofts)

MÜLLER, W. et al. eds: Rheumatoid arthritis (Acad. Press)

NEEDHAM, D. M.: Machina carnis: the biochemistry of muscular contraction and its historical development. (Cambridge Univ. Press)

NEU, H. C. ed.: Changing patterns of bacterial infections and antibiotic therapy. (Excerpta Medica)

NICHOLS, R. L. ed.: Trachoma and related disorders caused by chlamydial agents. (Excerpta Medica)

PAGE, I. H. & McCUBBIN, J.: Renal hypertension. (Year Bk. Med. Publ.)

PALMER, E. D.: Upper gastrointestinal hemorrhage. (Thomas)

### BRISTOL MEDICO-CHIRURGICAL SOCIETY

The Annual General Meeting was held in the Medical School on 11th October 1972. Dr. N. J. Brown showed pathological specimens and Dr. G. R. Airth demonstrated skiagrams. The retiring president, Dr. C. C. Morgans resigned the chair to his successor, Dr. James Macrae who delivered his presidential address on the subject of Edward Jenner; this appears on page 23 of this number of the Journal. Officers were elected and reports were received from the Hon. Secretary, Hon. Treasurer and Hon. Editor of the Journal who explained that publication was likely to fall somewhat behind owing to temporary lack of funds. It was hoped however that recovery of punctual appearance of the Journal would be achieved in time. Mr. H. L. Shepherd was elected an Honorary Member of the Society.

The Annual Clinical Meeting was held at the Radiotherapy Centre on 8th November. A discussion was held on the place of the Centre in the Medical Community and a buffet supper was provided by courtesy of the Board of Governors of the United Bristol Hospitals.

The meeting on 13th December took the form of a Symposium on "Dialysis and Transplantation in the Management of Chronic Renal Failure", the speakers being Dr. J. C. Mackenzie, Dr. G. H. Tovey and Mr. H. J. O. White. Dr. C. R. Tribe showed pathological specimens and Dr. J. B. Penry showed skiagrams.

At the meeting on 3rd January 1973 a most interesting address entitled "The Way Ahead" was given by Sir George Godber, K.C.B., Chief Medical Officer of the Department of Health and Social Security. Pathological specimens were shown by Dr. D. H. Johnson and skiagrams by Dr. J. L. G. Thomson.

The Fifth Meeting was held on 14th February when Mr. G. J. Hadfield, Consultant Surgeon, Aylesbury, spoke on "Benign Diseases of the Breast". Dr. R. Blight showed pathological specimens and Dr. J. Roylance demonstrated skiagrams.

At each meeting in the Medical School the medical librarian, Mr. B. P. Jones provided his usual fascinating display of medical books.

### UNIVERSITY OF BRISTOL

### EXAMINATION RESULTS AND DISSERTATIONS APPROVED

**M.D.**

Sandra Mary EGAN
Margaret Hilary MORGAN
Jagdish Chandra CHAWLA
John Stuart MORRIS
Richard Andrew MOUNTFORD

**Ch.M.**

Anthony HINCHCLIFFE

**Ph.D.** (Faculty of Medicine)

John Edward TYLER
Hubert Neil NEWMAN
Angela Mary PALMER
Farshid TOOFANIAN

**M.B., Ch.B.**

Richard Thomas JOHN
Christopher LEE
Paul Vincent McALLISTER

### UNIVERSITY OF BRISTOL

### *Faculty of Medicine*

The University grants the degrees of Bachelor of Medicine and Surgery (M.B., Ch.B.), Master of Surgery (Ch.M.), Doctor of Medicine (M.D.), Bachelor of Dental Surgery (B.D.S.), Master of Dental Surgery (M.D.S.), and Bachelor of Veterinary Science (B.V.Sc.), as well as diplomas in Public Health (D.P.H.), and in Dental Surgery (L.D.S.).

Clinical instruction is provided in the United Bristol Hospitals and in some of the Hospitals in the Bristol area under the control of the South Western Regional Hospital Board. Mechanical and clinical instruction in Dentistry is carried out in the University's Dental Hospital and School.

INCLUSIVE FEES

| | |
|---|---|
| For the M.B., Ch.B. curriculum ... ... | £87 per annum |
| For the B.D.S. curriculum ... ... ... | £87 per annum |
| For the L.D.S. curriculum ... ... ... | £87 per annum |
| For the B.V.Sc. curriculum ... ... .. | £87 per annum |

---

**For additional particulars apply to**

**THE REGISTRAR, THE UNIVERSITY, SENATE HOUSE, BRISTOL, 2**

